# Improving obesity treatment through online motivational support in primary care^☆^

**DOI:** 10.1016/j.obpill.2025.100191

**Published:** 2025-07-01

**Authors:** Jessica Stockham, Shannon Harris, William Berard

**Affiliations:** a West Georgia Internal Medicine, 706 Dixie St. Suite 300, Carrollton, GA, 30117, USA; b University of South Alabama College of Nursing, Mobile, AL, USA; c West Georgia Internal Medicine, Carrollton, GA, USA

**Keywords:** Obesity, Primary care, Digital interventions, eHealth, Social media

## Abstract

**Background:**

Obesity is a complex, chronic disease requiring time-intensive, multifaceted management strategies that are often difficult to implement in primary care. This quality improvement project aimed to evaluate whether the integration of an online motivational support group via Facebook, in addition to routine monthly office visits, could enhance obesity treatment outcomes and patient motivation in a primary care setting. As a nurse practitioner driven project, nurse practitioners have the training to make significant improvements in the area of obesity treatment. Nurse practitioners are key in providing patients with education and tools to help patients lose weight and maintain successful weight loss (Fruh, 2017) [1].

**Methods:**

This was a quality improvement project using a pre-post design conducted at a private outpatient internal medicine clinic. Adult patients (n = 68) with a BMI ≥30 or ≥25 with comorbidities were enrolled. Participants joined a Facebook-based support group and received standardized education and monthly in-person follow-up for 12 weeks. Outcomes measured before and after the intervention included weight, BMI, waist circumference, and responses to the Weight Efficacy Lifestyle Questionnaire-Short Form (WEL-SF). Paired t-tests were used for statistical analysis.

**Results:**

Statistically significant improvements were observed in weight (−9.46 lbs, *p* < 0.0001), BMI (−1.91, *p* < 0.0001), and waist circumference (−1.87 inches, *p* < 0.0001). WEL-SF scores improved significantly in 7 of 8 domains, with an average increase of 11.8 %, indicating enhanced self-efficacy and motivation for healthy eating behaviors.

**Conclusion:**

The integration of an online motivational support platform with monthly in-office visits significantly improved physiologic outcomes and patient self-efficacy in managing obesity. These findings support the feasibility and potential benefit of low-cost digital support tools in augmenting outpatient obesity care, particularly in settings with limited resources. Further research should explore the impact of engagement level and long-term outcomes.

## 1 Introduction

Obesity is an uncontrolled epidemic in the United States that is detrimental to the health and well-being of patients [1]. According to the CDC, approximately 41.9 % of adults were obese in 2020 which is an increase of about 10 % over the last 20 years [2]. At West Georgia Internal Medicine, the clinic mirrors the statistics of the country with approximately 42 % of patients having a BMI of 30 or greater. While the providers within the clinic do offer weight management services, there is no standardized education provided to patients and motivational support outside of routine office visits is lacking. Referrals are available to dieticians that practice within the area, however, most patients wait an average of 3 months to have an initial consult with a dietician. This lack of standardization and coordination prevents patients from receiving the obesity treatment that is needed for long term success.

Obesity is associated with co-morbid conditions such as hypertension, type II diabetes mellitus, heart disease, and cancer to name a few [3] Lowering body weight and BMI can help reduce the risk of these diseases and reduce the burden of these illness on the community. Obesity not only affects physical health but can also be detrimental to mental and cognitive health as well. In a population based study of 28,836 participants, body weight, waist circumference, BMI, and relative fat mass were all higher in individuals with depression [4]. This suggests a link between depression and obesity. There is also evidence to suggest that obesity may be linked to decreased cognitive function in adults over 60 [5].

Moreover, obesity not only increases patients' risks of serious health problems but can also be viewed as a disadvantage socioeconomically with patient's suffering from obesity often making less money than their normal weight peers [6]. The financial costs of obesity also affect the healthcare system. Medical care for adults with obesity was 100 % greater than adults with a normal weight in the United States in 2016 [7]. According to the CDC, the cost of obesity in 2019 was 173 billion dollars. These are serious physical and financial consequences that support the need to urgently address this problem within the practice.

Obesity management is thoroughly discussed in the literature and there is ample evidence on this topic. The use of technology and the internet has been valuable in the approach toward obesity treatment. A systematic review found that digital interventions, especially in low resource environments, have been effective at improving health and patient outcomes [8]. The use of the internet allows patients to have greater access to education and support from their health care provider while promoting a healthy lifestyle.

Additionally, it is important to consider the approach to obesity management in the outpatient clinic. Patients with clearly established goals and sustained motivation have better weight loss outcomes as determined by one systematic review of 294 individual accounts [9]. Calorie trackers and apps can be helpful tools for patients to keep them on track with their goals. Identification of intrinsic and extrinsic motivators is also crucial for long term success. In a quantitative, randomized control trial 105 adults with obesity achieved positive results in a self-administered internet based intervention [10]. This intervention provided patients with different learning tools to promote lifestyle changes to encourage weight loss. A separate systematic review and meta-analysis of randomized control trials also found that patients responded well to web based interventions for the treatment of obesity. Web based interventions with the most success included personalized feedback from healthcare providers and the development of self-regulation skills [11].

Increasing physical activity is a valuable tool in weight management and waist circumference reduction. Many patients experience barriers to increased physical activity as described by one systematic review. The majority of patients with obesity described lack of motivation, physical pain, and lack of time as significant barriers to physical activity [12]. Better understanding of patient barriers can help providers provide targeted motivation techniques to help patients be more successful. The type of physical activity incorporated into a weight management program is also significant. A systematic review of 114 trials, found that resistance exercises combined with caloric restriction were the most effective at reducing body fat [13]. One quantitative systematic review and meta-analysis found that in older adults with obesity, an exercise routine of combined aerobic and resistance exercise was effective at helping individuals lose weight without losing too much muscle mass [14]. This study only examined adults from ages 50–70 which could limit its applicability to patients of a younger age.

## 2 Methods

Participants in the project were patients of West Georgia Internal Medicine in Carrollton, GA. Participation in the project was voluntary and offered to patients as part of the current weight management services. Participants were provided with a written document outlining a description of the project and how their anonymized data would be collected and used. Included participants were ages 18 and up with no restriction on sex. Patients were required to have a BMI of 30 or greater or a BMI of 25 or greater with at least one weight related co-morbidity such as hypertension. Patients were excluded from the project if they were less than 18 years of age, on hospice care, have active cancer, receiving cancer treatment, untreated endocrine cause of obesity, pregnancy, current bone fracture, cognitive impairment, or diagnosis of anorexia or bulimia. A minimum of 50 participants was desired for the project.

The project was conducted at West Georgia Internal Medicine in Carrollton, GA. The clinic is a private, outpatient clinic with seven physicians and one nurse practitioner. There were no identified issues in the setting that could potentially affect the project.

The physiologic measurements obtained in the project were height, weight, and waist circumference. The Weight Efficacy Lifestyle Questionnaire Short Form (WEL-SF) was also utilized. The Weight Efficacy Lifestyle Questionnaire-Short Form (WEL-SF) is a self-administered tool that was developed to allow clinicians to measure patient confidence in controlling their eating behaviors [15]. The questionnaire includes 8 challenging situations in which patients rate their confidence level from 0 to 10 on their ability to control their eating. This provides a total score of 0–80. The validity of the WEL-SF is supported by lower scores being associated with unhealthy eating behaviors such as binge eating, food addiction, and night eating syndrome while higher scores were associated with healthier behaviors such as exercise and increased motivation [15]. In one study of 1750 patients, the WEL-SF has a Cronbach's alpha of 0.92 demonstrating excellent reliability [15]. This reliability remained consistent for each of the 8 scenarios of the questionnaire when they were evaluated individually with Cronbach's alpha ranging from 0.90 to 0.92 [15].

The project was conducted from August 2024 to December 2024. Participants were scheduled for an office visit in August. During the office visit, height, weight, and waist circumference was collected by the medical assistant. Medical assistants in the office were previously trained on how to collect this data. The collected information was documented in the patient's electronic medical record. At the office visit, the medical assistant provided the patient with the WEL-SF questionnaire. The questionnaire was self-administered by the patient and the completed questionnaire was returned to the primary investigator. The patient was provided with links to access the online support group through Facebook. The patient education content included information on how to begin a calorie restricted diet by reducing their caloric intake by 500 calories per day and starting an exercise routine. At the end of the visit, the patient was rescheduled for monthly follow appointments. At the 3 month follow up, height, weight, and waist circumference were measured again by the medical assistant for comparison. The WEL-SF questionnaire was also re-administered.

This project was exempted from IRB review by the University of South Alabama Institutional Review Board on July 23, 2024, and assigned as IRB Protocol: 24–353. The University of South Alabama IRB determined that this project did not meet the definition of human subjects’ research as defined in 45CFR46.102(e)(1). The project was deemed exempt from IRB approval or review as a quality improvement initiative. As such, written informed consent for this project was not necessary. The University of South Alabama Institutional Review Board is located at 307 N. University Blvd., AD 200, Mobile, AL 36688-0002.

## 3 Statistical methods

All collected data was organized in an Excel spreadsheet and analyzed using statistical software JMP Pro v. 18.0.2. Pre- and post-survey outcomes were matched using a unique identifier. All graphs were created using Excel. All numerical data was summarized using mean and standard deviation. Mean outcomes from pre- and post-intervention were compared using paired t tests. Results were considered significant at 5 % error rate.

## 4 Results

Pre-post data was available on 68 participants. Difference in outcome was computed as post-pre. A positive % change indicates post-intervention outcome is higher than the pre-intervention outcome, and a negative % change indicates post-intervention outcome is lower than the pre-intervention outcome.

From pre-to post-intervention period, a statistically significant decrease was observed in weight (221.06–211.60, paired *t*-test, p < 0.0001), waist circumference (44.32–42.45 in, paired *t*-test, p < 0.0001), and BMI (35.50–33.59, paired *t*-test, p < 0.0001) Fig. 2.

**fig 1:**
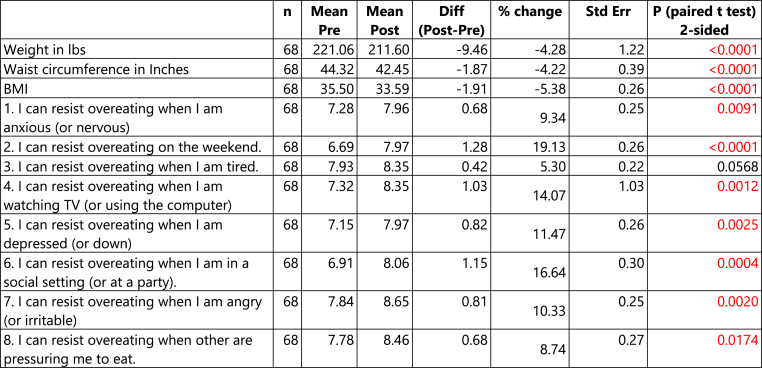

Except for question 3, all other questions in the WEL-SF survey resulted in an average significant increase in the outcome from pre-to post-survey. Even question 3 resulted in an increase in the mean outcome from pre-to post-survey, however it was not statistically significant (fig 1, fig 3).

In summary, patients lost significant weight, waist circumference, and BMI. They were also able significantly to resist overeating under different circumstances, except when tired.

## 5 Discussion

**fig 2:**
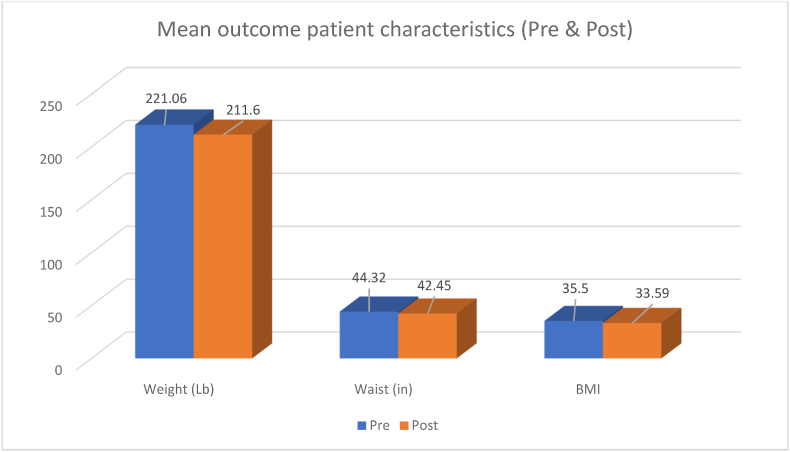

**fig 3:**
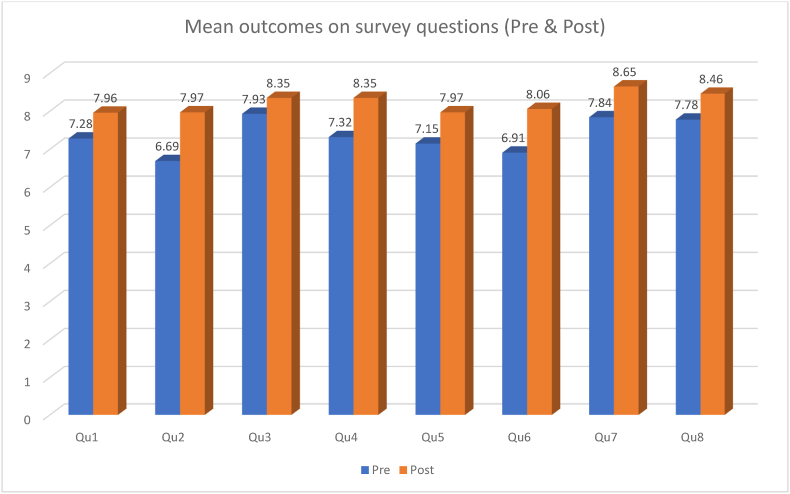

This study was able to demonstrate successful reduction in weight, BMI, and waist circumference over the course of 12 weeks in an outpatient primary care clinic with a total of 68 participants through monthly in office follow ups paired with an online Facebook support group. Overall, patients lost 4.28 % of body weight as well as a 4.22 % decrease in waist circumference and a 5.38 % decrease in BMI. Moreover, patient scores on the WEL-SF survey improved by an average of 11.8 %. These results indicate improvement in weight management self-efficacy and motivation (fig 1, fig 2, fig 3).

All of the participants were compliant with scheduled office follow ups, however, patient engagement in the online support group was minimal. Patients regularly viewed published content but rarely engaged in discussion. Further research is needed to compare whether weight reduction is increased with higher patient engagement in online support groups. It is also reasonable to consider that monthly in person follow ups with patients aided in successful weight loss and increased motivation.

## 6 Limitations

This study encountered several limitations during the research process. Several patients declined participation in the study due to concerns over social media and privacy issues. Some patients also declined because they did not have a Facebook account and did not desire to create one. At least one patient declined to participate due to lack of internet access. The study was also somewhat limited by internet literacy in patients. There were some participants who were eager to participate but did not know how to post within the Facebook support group. Additionally, the study was limited by a low sample size of 68. The geographic location is also a limitation of the study as it only includes residents in the West Georgia area and results may vary in other geographic locations.

## 7 Prior studies

No prior studies have been completed by investigators on this topic.

## 8 Conclusions

While treatment of obesity continues to be challenging for health care providers working in primary care, providing patients with online support in addition to routine office visits is an opportunity to improve patient outcomes and increase motivation. Incorporating online platforms into practice provides patients with unlimited access to helpful resources and education outside of the clinic. This has the potential to save health care providers time on in office education and focus more on individualized counseling. eHealth remains to be a growing field of study with opportunities for new developments and research in the future.

## 9 Clinical takeaway messages

• Integrating online motivational support with routine primary care follow-ups can significantly enhance weight loss outcomes and improve patient self-efficacy.
• Even low-engagement digital tools, such as a passive Facebook support group, can contribute meaningfully to obesity management in outpatient settings.
• Standardized education combined with digital access to resources may improve continuity of care and reduce barriers to long-term weight management.

## Ethics statement

This project was exempted by the University of South Alabama Institutional Review Board and deemed as a quality improvement initiative not requiring written consent from participants.

## Author contributions

Jessica Stockham served as the principal investigator, developed, and implemented the project, and conducted the data analysis. Shannon Harris contributed to project development and provided critical revisions to the manuscript. William Berard served as a mentor and provided guidance throughout project development and implementation. All authors reviewed and approved the final manuscript.

## AI usage statement

Artificial intelligence tools were not used in the writing, editing, data analysis, or any other aspect of this manuscript. All content was developed and prepared solely by the listed authors.

## Funding statement

There was no funding received for the completion of this quality improvement project.

## Conflicts of interest

There are no known conflicts of interest.

## Abbreviations

WEL-SF: Weight Efficacy Lifestyle Questionnaire-Short Form
CDC: Centers for Disease Control
